# Mini review: The role of sensory innervation to subchondral bone in osteoarthritis pain

**DOI:** 10.3389/fendo.2022.1047943

**Published:** 2022-12-20

**Authors:** Michael Morgan, Vida Nazemian, Kate Harrington, Jason J. Ivanusic

**Affiliations:** Department of Anatomy and Physiology, University of Melbourne, Melbourne, VIC, Australia

**Keywords:** subchondral bone, sensory innervation, sensitization, sprouting, osteoarthritis pain, neuropathy

## Abstract

Osteoarthritis pain is often thought of as a pain driven by nerves that innervate the soft tissues of the joint, but there is emerging evidence for a role for nerves that innervate the underlying bone. In this mini review we cite evidence that subchondral bone lesions are associated with pain in osteoarthritis. We explore recent studies that provide evidence that sensory neurons that innervate bone are nociceptors that signal pain and can be sensitized in osteoarthritis. Finally, we describe neuronal remodeling of sensory and sympathetic nerves in bone and discuss how these processes can contribute to osteoarthritis pain.

## Introduction

Osteoarthritis (OA) is a progressive degenerative disease of the articular cartilage that impacts on surrounding synovial tissues and bone, and is characterized by swelling, stiffness, and pain. Pain is the most debilitating aspect of OA, and both the onset and severity of pain are key factors that lead those who suffer OA to seek medical advice (1). While there is a justified focus on finding disease-modifying treatments that stop or slow the progression of OA, we can also make significant improvements to quality of life and economic burden by treating the underlying pain.

Cartilage is aneural, but the soft tissues and the subchondral bone around joints are richly innervated by sensory nerve endings that respond to noxious stimuli, can be sensitized by inflammation, and are relevant to the pathophysiology of OA pain (2–26). However, because OA has in the past mostly been considered a joint disease, and because the innervation of soft tissues of the joint are easily accessible in animal models, there has been significant focus on the role of the nerves that specifically innervate the articular tissues of the joint. There have already been a number of excellent reviews that summarize this literature (25, 27–31). In this review, we will instead provide a contemporary overview of the pathogenesis of pain in OA, with a particular focus on the role of the sensory innervation of the subchondral bone, which is likely to be important particularly in late-stage disease when there is significant damage to the bone surrounding osteoarthritic joints.

## Role of the innervation of synovial tissues and joint capsule in and around the joint

There have already been a number of excellent reviews that summarize the role of the articular tissue in the literature (25, 27–31). Articular nociceptors innervate the joint capsule and synovium, respond to noxious mechanical stimulation and known algesic substances applied to the joint, and can be sensitized by inflammatory mediators released in OA (3–8, 11–13). In studies of humans with OA, inflammatory mediators released in the inflamed synovium provoke pain, and there are a number of studies that report an association between knee pain and synovitis (32–34). However, while it is clear from radiographic evidence that joint damage predisposes to pain, there is little relation between the severity of joint damage and the pain experienced (35–38). In a study of 58 patients with OA and 33 pain-free controls, there was no association between the *extent* of synovitis and the *degree* of pain (39). These findings suggest that the activation of articular nociceptors as a result of joint damage and/or inflammation may not be the main contributor to the severe pain experienced by patients with OA.

## Clinical evidence for the involvement of the subchondral bone in the pathogenesis of OA pain

There is a growing body of evidence for the involvement of the subchondral bone in the pathogenesis of OA and OA pain (29, 40, 41). When the articular cartilage breaks down with the progression of OA, subchondral and synovial compartments become increasingly continuous, and histopathological changes in the subchondral bone (including microdamage, bone marrow lesions, and bone cysts) emerge (42, 43). These histopathological changes occur during the most debilitating stage of OA when pain is poorly controlled. In a cross-sectional observational study of 401 patients with knee OA, 351 patients reported painful knees and 50 patients reported no pain. Subchondral bone lesions were noted in 77.5% of those that reported knee pain, but in only 30% of those without knee pain (44). Importantly, the size of the bone lesion was independently associated with pain (44). This association was also observed in another cross-sectional study of over 1400 patients, where it was reported that the size of bone marrow lesions correlated specifically with weight-bearing pain (45). In advanced OA, severe pain may also be a result of raised intraosseous pressure in the bones around joints (46–51). In some of these cases, the increase in pressure has been associated with pain which can be relieved by fenestration, suggesting that increased pressure in the marrow cavity produces pain.

Interestingly, several clinical studies have tested whether bisphosphonates, a group of drugs that slow bone loss by reducing osteoclast activity, can reduce bone marrow lesion size and also OA pain. A randomized controlled trial of 60 patients with OA found that patients given four intravenous doses of neridronate had reduced bone marrow lesion size and reported less pain (52). A similar result was seen in a clinical trial of 59 patients given a different bisphosphonate, zoledronic acid (53). Patients with knee OA displayed reduced bone marrow lesion size and reduced pain at 6 months after a single infusion with zoledronic acid compared to placebo (53).

Together, these findings highlight a clear link between damage to the subchondral bone and pain. This role appears to be particularly important in late-stage OA, when there is cartilage breakdown, and provides evidence that changes to the function of nerves that innervate the subchondral bone may drive pain during this late stage of disease.

## Sensory innervation of subchondral bone

There is a long history of work documenting the innervation of the bone (9, 54–61). Nerves can be found entering the bone through the nutrient foramina, Haversian canals, the osteochondral (OC) junction, and at the attachments of the synovial membrane (23, 62–65). They branch extensively in the periosteum, bone marrow, and subchondral bone associated with synovial joints, where they end as unencapsulated, free fiber nerve endings (23, 60, 66). The bone is a highly vascularized structure, and most nerves found within the bone run with the vasculature (63, 65, 67), so many authors suggested they had a vasculature function but did not comment further. The advent of retrograde tracing in combination with immunohistochemistry has provided clear evidence that nerves within the bone are of both sensory and autonomic origin, and those that are sensory predominantly function as nociceptors (68, 69).

Pain is transmitted by two main classes of peripheral nociceptors (70). Small-diameter myelinated sensory neurons, known as Aδ nociceptors, transmit fast, intense pain, while small-diameter unmyelinated sensory neurons, known as C nociceptors, encode slow, aching pain. In the dorsal root ganglia (DRG), the soma of peripheral sensory neurons that innervate the medullary cavity, trabecular bone, and the periosteum are almost exclusively small-diameter myelinated (neurofilament rich) or unmyelinated (neurofilament poor) neurons (10, 15–17, 19, 21). They express markers characteristic of nociceptive neurons, with rodent studies finding that approximately half express calcitonin gene-related peptide (CGRP), two thirds express tyrosine receptor kinase A (TrkA), a quarter express Substance P, almost half express transient receptor potential vanilloid 1 (TRPV1), and some bind isolectin B4 (10, 16, 17, 20, 68, 71, 72). Both peptidergic and non-peptidergic bone-projecting nociceptors have been identified in DRG on the basis of various combinations of these markers (10, 72), and these molecular phenotypes are likely maintained in their peripheral nerve terminals in the bone (23, 66, 73, 74). Thus, it is clear that sensory neurons that innervate the bone have a morphology and molecular phenotype consistent with a role in nociception.

Until recently, there were few published studies recording the response of peripheral sensory neurons to noxious stimulation of the bone marrow (75, 76). These studies used anesthetized dogs to make whole-nerve recordings from branches of a nerve that innervates the tibial marrow cavity. Application of noxious mechanical and chemical stimulation to the marrow cavity evoked an increase in whole-nerve activity, but no attempts were made to characterize the response of individual bone marrow nociceptors to different types of noxious stimuli. More recently, an *in vivo* bone–nerve preparation was developed to record the activity of nerves that innervate the tibial marrow cavity of rats (18, 20). Electrophysiological recordings were made from a small branch of the tibial nerve, proximal to its entry into the tibia, in response to increasing intra-osseous pressure (to provide noxious mechanical stimulation), or through the application of noxious chemical stimuli, directly to the marrow cavity. Single bone nociceptors were isolated from the whole-nerve recordings with spike discrimination software providing unprecedented insight into the function of single sensory neurons that innervate the bone. This approach has been used to show that single sensory neurons that innervate the bone have conduction velocities consistent with Aδ and C nociceptor classifications, can be activated and/or sensitized by inflammatory mediators and known algesic substances directly applied to bone, and can respond to noxious increases in intraosseous pressure (16–22). Importantly, many of the inflammatory agents used in these electrophysiological recording studies also produced altered pain behavior when applied within the bone, providing a clear link between the altered function of sensory neurons that innervate the bone and pain (20–22). These physiological data provide strong evidence that sensory neurons found in bones have a role in nociception.

## Activation and sensitization of bone nociceptors in OA

**Figure 1:**
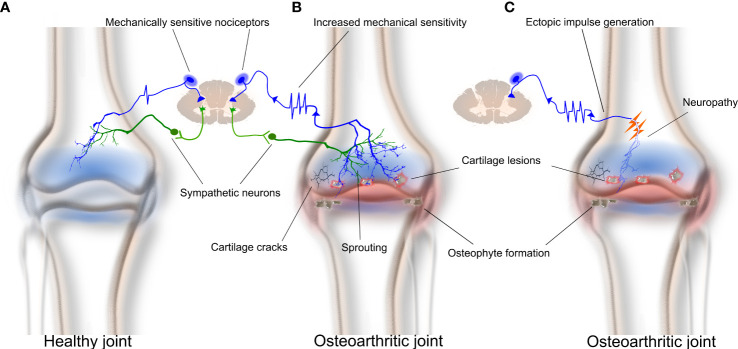
The subchondral bone contributes to osteoarthritis pain through mechanical sensitization, sprouting, and/or neuropathy of bone nociceptors. Osteoarthritis results in joint inflammation, reduced synovial space, breakdown of cartilage and cartilage lesions, osteophyte formation, and subchondral bone lesions and sclerosis. **(A)** A healthy bone with typical sympathetic and nociceptor innervation. **(B)** Subchondral bone remodeling in advanced osteoarthritis can result in increased neuronal sprouting of bone nociceptors and sympathetic neurons within bone. Sprouting sensitizes peripheral sensory neurons and makes them hyperexcitable. **(C)** Neuropathy of bone nociceptors may also contribute to osteoarthritis pain through generating ectopic spontaneous discharge.

Whilst there have been many high-quality studies that have explored mechanisms related to how OA affects the function of articular afferent neurons (13, 28, 77–80), there has been only a single report of how OA specifically affects those that innervate the surrounding bone (81). The latter study applied the *in vivo* bone–nerve preparation described above to make electrophysiological recordings of the nociceptors that innervate the subchondral bone (by recording the nerve to the rat tibia), or the articular tissues around the knee (by recording from the medial articular nerve), in a rat model of monoiodoacetate (MIA)-induced OA. MIA-induced OA is a robust and reliable animal model of OA that results in rapid changes in weight-bearing within a day post-injection, and which continue beyond 28 days (81–83). It is characterized by an early inflammatory response in the joint that is obvious in the first week (early-stage OA), and significant cartilage breakdown that predisposes to the inflammation and damage of the subchondral bone later in disease progression (late-stage OA) (81, 82). The authors showed that there were significant changes in the function of knee joint nociceptors, but not bone nociceptors, early in the progression of OA (day 3 post-MIA injection), when there was histological evidence of inflammation in the joint capsule, but no damage to either the articular cartilage or surrounding subchondral bone. The changes in articular nerve function noted in early MIA-induced OA included increased spontaneous activity, decreased thresholds for mechanical activation, and increased discharge frequencies in response to mechanical stimuli compared to animals without OA (81). Changes in the function of bone nociceptors occurred later in the progression of OA, when there was histological evidence of damage to the articular cartilage and surrounding subchondral bone. The changes in bone–nerve function noted at this later stage of MIA-induced OA included decreased thresholds for mechanical activation of Aδ bone nociceptors, and increased discharge frequencies for both Aδ and C bone nociceptors during mechanical stimulation. These data are the first to show that the progression of OA pathology from the joint into the subchondral bone is accompanied by functional changes to the nociceptors innervating the subchondral bone, and could account for the increased pain in OA patients later in the disease when there is pathology in the bone surrounding the joint (**Figure 1**).

Most of this work has been in MIA-induced OA. There have been a number of studies that have explored the temporal characteristics of pain associated with histopathological changes in the bone in alternative models, including partial meniscectomy and destabilization of the medial meniscus (30, 84–89). However, none of these have documented mechanisms by which the nerves in the bone contribute to this pain.

The findings of sensitization of nerves that innervate the bone cited here are important because they highlight the need to target the peripheral nociceptors that innervate the bone, in addition to those that innervate the joint, for therapeutic benefit in late-stage OA to be realized.

## Neuronal sprouting in bone and OA pain

Neuronal remodeling is a phenomenon that can occur as a result of tissue damage and can result in hyperalgesia, allodynia, and ectopic firing (90–94). One type of neuronal remodeling is neuronal or axonal sprouting, where axons from undamaged neurons in neighboring areas sprout into the damaged tissue region (95). There is significant evidence that sprouting of both nociceptors and sympathetic neurons promote pain in and around joints. In a painful animal model of experimental inflammation, injection of Complete Freund’s adjuvant into the joint resulted in the sprouting of sympathetic and sensory neurons into the synovium, and the blockade of nerve growth factor (NGF) signaling reduced both sympathetic and sensory neuron sprouting and pain behavior (96).

There is also evidence of increased innervation in the subchondral bone marrow of femoral heads taken from patients with OA compared to those without (97). In a more recent study, increased sensory and sympathetic innervation was observed at the OC junction, and in the bone marrow and osteophytes, of patients with tibiofemoral OA (62). These findings are supported by animal studies showing that both nerve fibers and vessels sprout through channels at the OC junction to get access to the inflamed joint cavity (98, 99) and provide a potential structural explanation for increased pain sensation in OA patients (**Figure 1**). Aso et al. (100) further examined the effect and pain outcomes of anti-NGF treatment on the innervation of subchondral bone in rats with meniscal transection-induced OA. Rats that received anti-NGF treatment had less sprouting of CGRP-immunoreactive nerves through OC junctions and displayed reduced pain behavior (100), suggesting a clear association between sprouting and OA pain.

## Neuropathy in bone and OA pain

There is emerging evidence for a role for neuropathy of nociceptors that innervate the bone in the pathophysiology of OA (28, 29, 101). One meta-analysis found that 23% of patients sampled reported neuropathic pain associated with their OA (102). Patients suffering from OA often use neuropathic descriptors to describe their pain, such as burning, numbness and pins and needles (103, 104), and application of therapeutics used to treat neuropathic pain in other conditions, such as gabapentin, often resolves the pain (105). In rats with MIA-induced OA, there is an increase in the number of DRG neurons that express ATF-3, a marker of sensory and motor neuron damage (106), and MIA administration results in the upregulation of other markers of neuronal injury and neuropathic pain in the DRG, including neuropeptide Y and interleukin-6 (107, 108).

One hypothesis for the cause of neuropathy in OA is damage to the subchondral bone in late-stage OA (109). It is possible that nociceptive nerve fibers become damaged when there is destruction of subchondral bone, such as that which is associated with bone marrow lesions and sclerosis (**Figure 1**). Damaged nerve axons are known to spontaneously and ectopically fire, and therefore can signal pain to the central nervous system (CNS) (110). Another potential mechanism is known as ‘cross talk’ or ‘ephaptic cross talk’ (111). This term refers to the phenomenon whereby regenerating nerve axons form synapse-like links with adjacent nerve axons following injury. The result of this process is abnormal nerve-to-nerve communication that can contribute to changes in the pain signals presented to the CNS.

Whilst it is possible that some of the physiological changes to the function of nociceptors reported are a response to the damage of peripheral nerve terminal endings in the subchondral bone in late-stage OA, a clear link between the two remains to be determined. It will be important in the future to determine how neuropathy affects nociceptors that innervate the bone, and its contribution to the pathogenesis of OA.

## Conclusions

The role of nerves that innervate bone in OA pain has been of increasing interest due to the relationship between pain and subchondral bone pathology. There is evidence that bone nociceptors contribute to OA pain in several ways including sensitization, sprouting, and neuropathy. Targeting nociceptors that innervate the bone may provide an effective strategy to treat OA pain.

## Author contributions

MM, VN, KH, and JI all contributed to literature searches and writing the manuscript. All authors have read and approved the final version of the manuscript.
